# Book Review

**Published:** 1936

**Authors:** 


### Reviews of Books

**William Budd.** By E. W. Goodall, O.B.E., M.D. Pp. 159. Illustrated. Bristol: J. W. Arrowsmith Ltd. 1936. Price 5s.—This small volume presents in very agreeable form an account of the family, surroundings, life and work of William Budd, for many years physician to the Bristol Royal Infirmary. To local medical men it has, perhaps, a particular appeal. Yet the whole of the profession should feel an especial interest in the account of the world's foremost epidemiologist. If there is a criticism of the book, it is that not sufficient emphasis is laid on the outstanding nature of Budd's work. He put forward the most revolutionary ideas—ideas that brought upon him the ridicule and contempt of the leading men of his day. He maintained that cholera, typhoid and, finally, consumption were "contagious," or as we should say "infectious diseases"! Also, that typhoid and typhus were different diseases, with distinct pathology and modes of transmission. But this was only a small part of his work: he set out to demonstrate his extraordinary ideas and, with particular reference to typhoid, he proved his case in the most careful, thorough and complete manner—long before the typhoid bacillus had been recognized. His best monument is his book on *Typhoid Fever*, published in 1873, nearly twenty years after the first demonstration of his thesis: and the fact that Budd's rules for the control of typhoid epidemics are in use, practically unchanged from their first form, all over the world to-day. Except that the really astonishing nature of Budd's original conceptions and demonstrations receive less attention than they merit, this very brief biography is excellent. It is written in a pleasant and readable style, contains several most interesting illustrations, and is well indexed.

**Health, Sickness and Psychology.** By Rev. R. W. Wilde, M.A., B.Sc. Pp. x., 202. Oxford University Press. 1936. Price 3s. 6d.—This is a really excellent little book. It is simple, readily intelligible and interestingly written. The author, who is a practising pyschologist, has set out in non-technical language the current views about the psychology of disease, and has sought to explain with great success how much disease of body is really due to more deep-seated trouble in the mind. His book has been read in proof by some physicians, and in deference to their views he has added some notes to some of the chapters. He is most careful to stress the need for a complete physical examination before psychological treatment is commenced, and is evidently accustomed to work in the closest collaboration and harmony with members of the medical profession. The reviewer gave the book to a lay reader absolutely ignorant of the subject to test its effect upon such a person. The result proved that the author has admirably succeeded in his task. If only we could persuade some of our patients to study this book and its lessons we might be able to cure more of them ! Although perhaps none of us would be prepared to endorse all that the author claims (would that insomnia were quite so easily cured !) yet this is a wise, understanding, helpful little volume, which can be safely recommended to the inquiring layman, and would be helpful to many a medical practitioner.

**Illustrations of Regional Anatomy. Section VI.: Upper Limb.** 42 Plates. Price 7s. 6d. **Section VII.: Lower Limb.** 52 Plates. Price 10s.—By E. B. Jamieson, M.D. Edinburgh : E. & S. Livingstone. 1936.—These two sections complete this very excellent series of *Illustrations of Regional Anatomy*, which now covers the anatomy of the entire body. The plates are beautifully presented, all of them being coloured and some being enhanced by having a black background. Separate plates are devoted to the bones, each bone being shown from several aspects and having the attachments of the muscles and the joint capsules and the position of the epiphyseal lines clearly marked. Moreover, the fact that in most of the drawings of the soft parts the outline of the underlying bones is shown by dotted lines is of distinct value. Although these illustrations would be useful to the student as an aid to dissection and as a substitute for the dissected part when that is not available, it would seem that their chief value is as an aid to the building up of a mental picture of the parts during and after systematic reading. The surgeon, too, might well find a brief study of the appropriate illustrations to be the easiest way of refreshing his memory of anatomical details for some unusual operation. The fact that each plate is easily removable has obvious advantages in this connection. It is perhaps logical that the dissections of the hand should be depicted in the " anatomical position," *i.e.* with the finger-tips towards the bottom of the plate, but many students will prefer to think of the various structures as they lie when he studies his own hand, viz. with the finger-tips upwards. It is, of course, a minor point and one that is easily rectified by the student himself if he so wishes. Incidentally, the illustrations of the synovial sheaths of tendons, both in the hand and in the foot, are admirable. The names used in the legends are those approved by the Anatomical Society at Birmingham in 1933.

**Diseases of Children.** Second Edition. By BRUCE WILLIAMSON, M.D., M.R.C.P. Pp. xii., 329. Illustrated. Edinburgh: E. & S. Livingstone. 1936. Price 10s. 6d.—The appearance of a second edition of this book will be welcomed by general medical practitioners and students who desire a quick reference book on children's diseases which can be easily carried in the pocket. Here he will find a short description of all the medical diseases he is likely to come across in practice, including the specific fevers and the commoner skin complaints. Its size necessitates briefness, but the inclusion of some very rare diseases could, we feel, be omitted, and accounts of more common complaints be amplified. The treatment of empyema, a difficult subject, is dismissed in a few vague lines. Lobectomy and postural drainage are not mentioned in the treatment of bronchiectasis, nor blood transfusions in hæmophilia. Some of the author's opinions, especially with regard to rheumatic heart disease, are open to criticism. The endocardium over the valve as a nidus for bacteria in this complaint has not been proved, nor is the view generally held to-day. Simple mitral regurgitation persisting for years is unlikely without stenosis, in view of the pathology of the disease and its associated fibrosis. The description of rickets is rather marred by the statements that the disease is due to lack of vitamins A and D, and that vitamin D is activated by ultra-violet rays. The chapters on infant feeding, functional nervous disorders and pneumonia are, however, lucid and to the point, and lists of causes of the more common alimentary disturbances in infants will be found to be very helpful. A good formulary is attached to the end of the book with short notes on the more important drugs.

**Legal Problems in Medical Practice.** By D. HARCOURT KITCHIN, Barrister-at-Law. Pp. 232. London: Edward Arnold & Co. 1936. Price 10s. 6d.—This is really a first-rate book and very readable. Most of us, of course, remember the articles of which it is comprised coming out in the *British*

*Medical Journal* in serial form. Inasmuch as the subjects dealt with are not as a rule included in medico-legal text-books they fill a gap in the library of a medical practitioner. The thorny question of negligence in its many aspects is dealt with clearly and well, and the cases cited from the law reports elucidate the difficult points in a very practical manner. The section in this book of special value and interest to the practitioner is the one describing the duties and difficulties of the medical referee. Included in this chapter also is a discussion on the vexed problem of fees and expenses in connection with giving evidence in court. This book thoroughly justifies its title, and can be cordially recommended to all practitioners of medicine who want sound advice on the legal problems of their professional lives.

**Home Care of the Mental Patient.** By Dr. ARIE QUERIDO. Pp. viii., 94. Oxford University Press. 1936. Price 2s. 6d.—This small book describes the methods which the author has found most beneficial in dealing with patients who are mentally afflicted but who are not suitable subjects for institutional treatment. He bases his views chiefly upon the good results obtained in the Netherlands, where domiciliary treatment is so largely practised. The various types of mental disorder are divided into four groups, and under each category suggestions are made as to the best form of management to be adopted, especial stress being laid upon the advisability of the relatives understanding the true nature of the illness if home-care is to be successful. Dr. Querido's views will be of special interest to members of the profession who have been looking for some modification in the law applying to home-treatment as advocated by the Board in its annual report for 1933. The book is well produced, and from every point of view a pleasure to read.

**Reports on Chronic Rheumatic Diseases. No. 2.** By C. W. BUCKLEY, M.D., F.R.C.P. Pp. x., 140. London: H. K. Lewis & Co. 1936. Price 10s. 6d.—The second volume of these reports maintains the high standard of excellence of the first. The volume appears in the same form of a series of reviews and original articles by different workers in the field of the chronic rheumatic diseases, with an appendix of brief abstracts of the most important of the foreign publications since the appearance of the first annual report. It is safe to say that this volume will be welcomed by all who enjoyed the authoritative quality of the earlier number. A special feature of this number is the admirable and comprehensive survey of recent American work on rheumatism and arthritis by the Secretary of the American Committee for the Control of Rheumatism. This masterful summary more than takes the place of the review of the American literature which formed a valuable part of the first volume. Other important features are (*a*) the stressing of the probable ætiological relationship between acute rheumatic manifestations and chronic rheumatoid or atrophic arthritis, and (*b*) the report of present investigations in this country into the physiology of normal joint tissues. There are, in addition, many other original articles and reports, the general standard of which is probably higher than that of the first number. As before, the Committee takes no responsibility for the views of the authors of the articles; but the selection of the material included in the two reports which have thus far appeared leaves no doubt in our minds that the Committee's work will inevitably advance our knowledge of the unsolved problems of ætiology and treatment of chronic rheumatic diseases.

**Operative Surgery.** By ALEXANDER MILES, M.D., LL.D., F.R.C.S., and D. P. D. WILKIE, M.D., F.R.C.S. Second Edition. Pp. xx., 632. Illustrated. Oxford University Press. 1936. Price 21s.—The first edition of this excellent work appeared in 1933, the second in October, 1936. It is written by nineteen surgeons of the Edinburgh school, and is the best small text-book of operative surgery published in Great Britain; there are, of course, some much larger tomes which fulfil a different purpose. For the student and the occasional surgeon at home or abroad it is entirely adequate, and the pure consultant will be wise to make himself well acquainted with it whatever else he may read. In the second edition the illustrations have been improved, and the text revised. Opinion will differ, of course, as to which operations shall be included and which left out. We should have been glad to see the operation to fix the tuberculous sacro-iliac joint by driving a bone graft through the ileum into the sacrum, as well as the Smith-Petersen procedure. The sections on cerebral surgery and operations on the stomach and gall-bladder are particularly good. Division of the scalenus anticus might well have been mentioned for the treatment of symptoms of pressure on the lower cord of the brachial plexus popularly but incorrectly attributed to "cervical rib." We are surprised not to find Harris's method of prostatectomy mentioned, also that cancer of the sigmoid is apparently to be treated by resection with end-to-end or side-to-side anastomosis without any form of external drainage to relieve the pressure, and that occlusive resection is not mentioned. Fortunately, Devine's method of decompression is appended as useful "for unfavourable cases."

**Cystoscopy and Urography.** By JAS. B. MACALPINE, F.R.C.S. Second Edition. Pp. xvi., 478. Illustrated. Bristol: John Wright & Sons. 1936. Price 30s.—This work deals with urological investigation in a complete and exhaustive fashion. One of the attractive features in it is the historical survey of the evolution of different methods of investigation with which many chapters open. Every detail in the mechanism of cystoscopes, the media and cystoscopic procedure is explained with clarity and precision. The need for delicacy and discretion in handling is brought home to the reader, and possible pitfalls are exposed. Full instructions are given for the use of the diathermy electrodes, both in bladder and prostate lesions. The instrumentation of ureteric calculi is fully discussed. There are excellent descriptions of the benefits and complementary relationship of ascending and excretory pyelography. In these, as in all branches af the subject, admirable illustrations are given, of which many are beautifully coloured. The treatise is written in careful didactic prose, and the author is at pains to explain the arguments for or against the various methods, and inspires confidence by his cautious manner of writing and careful reasoning.

**Paget's Disease of the Nipple.** By KEITH INGLIS, M.D., Ch.M. Pp. xii., 234. Illustrated. Oxford University Press. 1936. Price 36s.—This is mainly, as the title indicates, an exposition of the author's views on the nature of Paget's disease of the nipple. These views, set out in summary form practically at the beginning of the book, are briefly that Paget's disease is a special variety of duct cancer commencing at the junction of a lactiferous duct with the epidermis, or in the duct near its outlet, and spreading from there both into the epidermis and backwards along the ducts. The first half of the book is concerned with a detailed consideration of a series of cases illustrating the arguments supporting this hypothesis, while the second deals with related conditions of the nipple, the breast and the extramammary skin. The volume is in clear print on excellent paper, and is profusely illustrated, mainly by photo-micrographs. These illustrations are remarkably good and well chosen. At the end of each chapter is a short summary of its contents, and the main conclusions and contentions are classified in the final chapter. There is a list of references, and the index appears to be satisfactory. It is obviously to the pathologist that the book will make its main appeal, but it has much of interest and value for the surgeon and for the dermatologist. For the pathologist the author's views are matters for discussion and argument, and time alone will decide on the merits of his hypotheses. For all, however, the book will always have this especial value, that here in one small volume are collected clearly-described and beautifully-illustrated examples of most of that group of lesions of the breast and its skin which has presented so many unsettled problems of ætiology and course.

**Favourite Prescriptions.** Edited by Sir Humphry Rolleston, G.C.V.O., K.C.B., M.O., F.R.C.P., and Dr. A. A. Moncrieff. Pp. 228. Eyre & Spottiswoode. 1936. Price 10s. 6d.—This book consists of favourite prescriptions from the large London general hospitals, the Dublin hospitals and the Edinburgh Royal Infirmary. Besides these, the pharmacopœias of five special hospitals in London are described: the Brompton Hospital for Consumption and Diseases of the Chest, the National Hospital for the Relief and Cure of the Diseases of the Nervous System, the Hospital for Sick Children, Great Ormond Street, St. John's Hospital for the Diseases of the Skin, the Central London Throat, Nose and Ear Hospital, and the Hospital for Tropical Diseases. A short history of each pharmacopœia is given, followed by the most popular prescriptions, which are set out in full. In most cases a few words are said about the symptoms which are particularly benefited by each mixture. The book is extremely readable, and should be of great value to the general practitioner by showing what combination of drugs, whether in mixtures, ointments, liniments or gargles, has been found by experience to be the most efficacious. It should also be of considerable interest to anyone prescribing for hospital out-patients. In the historical notes the dates of the first pharmacopœias of the hospital are given and the various omissions and additions commented on. The origin of many famous prescriptions is brought to light, such as Scott's dressing (Ung. hydrarg. co.) and Sir Jonathan Hutchinson's pill (hydrarg. et ipecac. co.) from the London Hospital, Gee's linctus (Linctus camph. co.) from St. Bartholomew's Hospital, and Ogle's drops (Linctus morph. hydrocyan.) from St. George's Hospital. A complete index is a very useful addition.

**Elementary Pathology.** By K. S. THOMPSON, M.R.C.S., L.R.C.P. Pp. viii., 74. Illustrated. London: H. K. Lewis & Co. Ltd. 1936. Price 10s. 6d.—Dr. Thompson has set out to provide a book which is to supply the basic reading matter for the elementary student of pathology as well as to act as a notebook for lecture and reading. It is not intended as a text-book and is, in fact, largely made up of blank pages (very glossy) for use by the student. The teaching of the elements of any subject is always the most difficult part of the lecturer's work, and this attempt at simplifying it is to be commended on these grounds. The illustrations are large and numerous for the size of the book, well-selected and, on the whole, fairly clear. The elementary character of the subject-matter is achieved not so much by the simplification of explanation as by condensation of the usual contents of the simple text-books. In this process much that others may regard as valuable has been lost, and the concentrate has come to contain elements which appear to be distortions of the usually accepted teachings. The arrangement of the subject-matter is along conventional lines, except that "Immunity" and "Changes in the circulating blood" are awkwardly interposed between the "Specific granulomata" and "Tumours," while "Ulceration" is strangely given a special chapter at the end of the book. A section on "Blood Transfusion" appears out of place in such a general and elementary volume. In view of the recommendation from the General Medical Council that the elements of pathology are to be included in the teaching of medical students during the year preceding their introduction to the clinical subjects it is probable that many teachers will welcome Dr. Thompson's book as the basis for this course, with the knowledge that subsequent lectures, etc., will enable the student to eradicate the misconceptions which such extreme condensation may have produced. In the preface it is suggested that the book may also be found useful for dental students, for nurses and for massage students, and it is probable that for these this book will fill a much-felt want.

**A Theory of Cancer and Mnemotherapy.** By RUDOLF ROOSEN, M.D., translated by C. F. MARSHALL, M.D., F.R.C.S. Pp. 75. London: Baillière, Tindall and Cox. 1936. Price 3s. 6d.—In this small volume of 75 pages the author expounds his theory that the cause of cancer lies in the development by the normal cell of "unlimited displacing power" as the result of the increasing of its permeability to Potassium by some agent the nature of which is obscured in the concept of "the engramm of a cancerous change." The therapeutics of cancer is simplified to a reduction of the "swelling pressure" of the cells. This is to be effected on lines similar to the treatment of chronic inflammation, and the author's recommendation for the purpose is Isamin Blue. In the second part of the book the principles and therapeutic potentialities of "the mneme," "the psychoids," and "the impulse psyche" are developed. Mnemotherapy is intended to be a rational combination of psychotherapy and medicinal measures aiming at regenerative stimulation. The book should be read by students of Cancer Research as an exposition of an unorthodox theory and therapy for which good results are already claimed, and by psychiatrists as a new and plausible explanation of some of the good effects of their methods of treatments. The general practitioner, for whom the book was ostensibly translated, will probably await the results of controlled experimental testing of these new tenets before applying them in daily practice.

---

### ADDENDUM, 1936, TO THE BRITISH PHARMACOPŒIA, 1932.

THE Addendum, which became available officially on 29th December, 1936, is a book of 132 pages, containing about 120 monographs, besides corrigenda of the B.P. 1932, appendices, etc. Its additions and amendments are pre-eminently those calculated to bring the 1932 B.P. up to date, and to render unnecessary the use of proprietary articles or at least those articles particularly associated with certain brand names.

Five new Antitoxins and Sera appear, two for Gas Gangrene (Œdematiens and Vibrioseptique), one for Staphylococcus and two for Pneumococcus (for types I and II.).

Three new Vitamin preparations appear, Acid Ascorbic (Vitamin C), Pulvis Vitamin B, Calciferol (Irradiated Ergosterol) and its liquor replace the Liq. Ergosterol. Irradiat. of the B.P. 1932, and finally Oleum Morrhuae becomes standardised for Vitamins A and D with a reduced dose, as is also the case with Liq. Calciferol.

While on this subject it is to be noted that the dose of Ferri. et Am. Cit. is increased to 20–40 grains as was suggested by Hurst, and Acriflavine, now accepted as a mixture of two acridine compounds, is assigned a dose $\frac{1}{2}$–$1\frac{1}{2}$ grains.

Chassar-Moir's work, culminating in the discovery and isolation of Ergometrine, received unusually rapid recognition by the inclusion of Ergometrine with doses for intramuscular and intravenous injection ; an interesting innovation, as the discovery of Ergometrine largely discounts the Acid Alcoholic Liquid Extract of the B.P. 1932, while rehabilitating the old Aqueous extract which was the stand-by of Gynæcologist and General Practitioner against all the theories of the Pharmacologists.

Modern physical aids to diagnosis are represented by Oleum Iodisatum, the official equivalent of Lipiodol—here one is inclined to question the advisability of a B.P. monograph giving entirely insufficient detail as to practical manufacture. Calcium medication is provided by the inclusion of Calcium Gluconate and a crystalline form of Chloride—$CaCl_2$. 6 $H_20$ representing 50 per cent. $CaCl_2$ for use in injections. One notices that the solubility in water of the gluconate (1–30) is only about a third that claimed by the makers of Calcium Gluconate (Sandoz).

Sodium thiosulphate is now included in the official list, a very necessary recognition of its use as routine treatment after the administration of organic arsenic, and also as a practical source of colloidal sulphur.

Modern organic arsenic preparations are seen in Tryparsamide. Mersalyl and its 10 per cent. injection provide an official prototype of Salyrgan. Chiniofonum for rectal administration as an antiseptic in amœbic dysentery and Histamine - Acid - Phosphate for subcutaneous injection in chronic rheumatism, indicate much greater provision for the needs of modern clinical medicine than ever before in a British Pharmacopœia.

